# CIRCADIAN TIMING OF THE IMMUNOMETABOLIC RESPONSE AFFECTS ALZHEIMER’S DISEASE PATHOLOGY

**DOI:** 10.1093/geroni/igad104.1678

**Published:** 2023-12-21

**Authors:** Jennifer Hurley

**Affiliations:** Rensselaer Polytechnic Institute, Troy, New York, United States

## Abstract

Though the predominant model of the biological mechanisms underlying ADRDs is based on the beta-amyloid (Aβ) hypothesis, evidence suggests that inflammation from resident immune cells is also a key component of the development of ADRD pathophysiology. A major biological regulator of inflammation is the circadian clock, the 24-hour molecular timekeeper that broadly regulates gene expression throughout mammalian tissues to synchronize biology to the Earth’s day/night cycle. Concordantly, chronic disruption of the circadian clock (CD) increases the risk for and severity of ADRDs. Our research has revealed that the circadian clock times metabolism, with the clock timing mitochondrial division which in turn times the response of mammalian macrophages to ADRD pathological factors. We show that subunits of the enzyme NOX2, a driver in the creation of reactive oxygen species (ROS) that uses metabolites to impart inflammation, are driven to oscillate in a 24 hr rhythm by the circadian clock in macrophages and microglia. Notably, oscillations in these NOX2 subunits are dysregulated when an organism undergoes CD, leading to an increase in the levels of ROS released by macrophages and microglia and elevating overall levels of inflammation. Given the apparent interconnection between inflammation, circadian rhythms, and ADRDs, an understanding of the circadian timing of the immune system could be exploited to develop treatments that alleviate ADRD symptoms, contribute to a potential cure, and prevent a population-wide increase in neurodegeneration due to a societal surge in CD.

